# Validity and Reliability of Rule Orientation Scale among Romanian Physicians—A Pilot Study

**DOI:** 10.3390/medicina59071217

**Published:** 2023-06-28

**Authors:** Maria Cristina Plaiasu, Dragos Ovidiu Alexandru, Bianca Hanganu, Codrut Andrei Nanu, Beatrice Gabriela Ioan

**Affiliations:** 1 Doctoral School, University of Medicine and Pharmacy of Craiova, 2 Petru Rares St., 200349 Craiova, Romania; cristina.plaiasu@umfcv.ro; 2 Department of Medical Informatics and Biostatistics, University of Medicine and Pharmacy of Craiova, 2 Petru Rares St., 200349 Craiova, Romania; 3 Legal Medicine Department, Faculty of Medicine, Grigore T. Popa University of Medicine and Pharmacy, 16 Universitatii St., 700115 Iasi, Romania; beatrice.ioan@umfiasi.ro; 4 Department No. 14 of Orthopedics, Anesthesia, and Intensive Care, University of Medicine and Pharmacy “Carol Davila”, 37 Dionisie Lupu St., 020021 Bucharest, Romania; andrei.nanu@umfcd.ro

**Keywords:** legal compliance, Romania, physicians

## Abstract

*Background and Objectives:* Legal compliance is influenced by several factors, including individuals’ attitudes about when breaking the law may be acceptable or justifiable. The rule orientation scale provides a measurement capable of predicting an individual’s offensive behavior, regardless of the legal punishment. The current research is the first that aims to evaluate the construct validity of the translated Romanian version of the rule orientation scale. *Materials and Methods*: A cross-sectional study was conducted online among Romanian physicians in Dolj County. A 12-item questionnaire previously validated in the United States was used for this study. *Results*: A total of 69 physicians responded to the survey with a mean age of 38.53 ± 8.28 and an average experience of 10.49 ± 8.27 years. Physicians were prone to adhere to the law and found only a few instances when legal breaches were acceptable. Nonetheless, they deemed it permissible to violate the law when they did not know its content. These attitudes were not affected by respondents’ ages, genders, numbers of years in practice, industries, or specialties. The internal consistency of the questionnaire was high (Cronbach’s α = 0.925). *Conclusions*: The rule orientation scale validated in the Romanian language can be used to determine conditions under which individuals find it acceptable to break the law.

## 1. Introduction

Individuals form social relationships based on their moral compass. Fairness is a concept determined by several internal elements [1]. Unethical behavior may bring public opprobrium, but if not sanctioned by legislation, it may go unpunished. Although in natural law theories, the duty to comply with a rule was typically attributed to moral obligations, promulgating the law was almost universally regarded as necessary [2]. In this context, even though ethics are at the heart of the legal system, individuals are compelled to observe the rule of law only from its promulgation, regardless of their moral beliefs.

Although anyone can occasionally break the law, some individuals repeatedly engage in unlawful behavior [3]. To address issues related to noncompliance with the law, it is important to understand its underlying causes.

Medical law is young and focuses on the rights and responsibilities of patients and physicians. The enforcement of medical law could result in physicians’ civil, disciplinary, and criminal legal liability.

Previous research has indicated an increasing trend in malpractice risk, with 16.1% of physicians in Romania being accused of medical malpractice by their patients [4]. Physicians from certain specialties, such as obstetrics and gynecology, emergency medicine, general surgery, and orthopedics and traumatology, are the most targeted by malpractice accusations [5,6].

Although medical courses have been taught in Romania for a few years, there is still an unmet educational need in this field. It is concerning to note that at a national level, nearly half of attending physicians and general practitioners did not comply with the law relating to confidentiality and patient data access, informed consent, discrimination, competency, and second medical opinion [7]. Noncompliance with the law in the medical profession can endanger patients’ health and safety while undermining trust in the healthcare system. While medical insurance is an essential mechanism in the healthcare system, it cannot protect physicians who violate the law [8]. In this context, it becomes important to observe the compliance issues related to the medical profession and to identify its possible roots.

Because of a series of factors, individuals are more or less prone to follow the law independently of the severity of the punishment or the social context.

Before the COVID-19 pandemic, no research had examined Romanian physicians’ attitudes toward the law. Previous nationwide retrospective studies indicated low legal compliance. Therefore, the authors assumed that the primary cause of physicians’ behaviors was a lack of legal knowledge and awareness, as no legal education programs were available for physicians at the time. Because legal knowledge can influence legal compliance, the lack of legal awareness was anticipated to be the leading cause of physicians’ conducts. Further research was conducted to explore physicians’ understanding of legal requirements, revealing that 90% of physicians were unaware that their practices did not comply with the law. In this context, it is critical to further investigate Romanian physicians’ views on violating the law. The study conducted by Adam Fine in 2016 created the rule orientation scale to evaluate how individuals perceive the appropriateness of breaking the law [9]. The rule orientation scale predicts potentially offensive behavior because highly rule-oriented persons identify fewer scenarios in which it is acceptable to breach the law. People who are low rule-oriented see more circumstances in which it is appropriate to transgress rules. The scale enables the assessment of potentially unlawful conduct without regard to the legal framework or context, whether it involves online rule breaking or offline activities, without considering the deterrent effect of the law.

The primary objective of this study is to validate and test the reliability of the rule orientation scale among Romanian physicians. In addition, the study aims to assess physicians’ attitudes toward breaking the law and investigate whether there are any differences in these attitudes based on various demographic factors, including specialties, ages, genders, years of experience in the profession, locations, and work systems.

## 2. Materials and Methods

A cross-sectional study was conducted on a convenience sample of physicians with the aim of validating the rule orientation scale and assessing their beliefs about breaching the law. To achieve this objective, we invited Romanian physicians from Dolj County to participate in an online survey designed for preliminary testing.

Our sample was drawn from all specialties, and we included physicians who passed the residency exam, regardless of their sector of practice, whether public or private. There were no exclusions based on any criteria, ensuring that our study was inclusive and representative of the physician population in the region.

In order to reach the participants, we utilized an online survey accessible via a Microsoft form. The survey was made available for a period of two weeks in February 2023, during which time participants were invited to complete it at their convenience. In order to obtain a comprehensive understanding of the participant’s background, we collected general demographic data such as their area of specialty, age, gender, years of professional experience, location, and work system. The information was collected through a completely anonymous questionnaire, and we ensured that no personal data were obtained or stored during the study. This approach was taken to protect the privacy and confidentiality of the participants.

Moreover, the study was conducted in accordance with the ethical guidelines and principles established by the Ethical Committee of the University of Medicine and Pharmacy of Craiova. Specifically, the study was reviewed and approved by the committee under reference number 47/10.02.2023. The research was conducted ethically and responsibly. Prior to participating in the study, all participants were provided with a detailed explanation of the research’s scope and objectives. Furthermore, participants were asked to provide written consent before completing the survey.

### 2.1. The Survey

To select an appropriate tool for assessing physicians’ attitudes toward breaking the law, we conducted a comprehensive literature review. After carefully considering several options, we ultimately decided to use the rule orientation scale, an English-language questionnaire that was previously validated in the United States [9]. The rule orientation scale was chosen for a variety of reasons. First and foremost, it was proven to be a trustworthy and valid instrument for assessing attitudes toward breaching the law. In addition, it was increasingly employed in prior studies and proved to apply to a vast array of settings and populations. The scale contains 12 items, and each item is evaluated on a 7-point Likert scale ranging from “strongly agree” to “strongly disagree”. Each item begins with the phrase, “It is acceptable to break a legal rule if”, followed by a scenario for which respondents were requested to assign a score.

Following the academic criteria, the questionnaire was validated in Romanian [10]. Initially, the questionnaire was translated from English to Romanian by a professional translator. The translation was then reviewed and debated by specialists, and some modifications were made to the initial Romanian version. Subsequently, a second review was conducted based on feedback from several physicians. Finally, two native-speaker translators re-translated the questionnaire back from Romanian to English, and the two versions were compared to ensure the accuracy of the translation. The final version of the translated rule orientation scale is presented in our study (Table 1). As per the original study, each item of the rule orientation scale began with the phrase “Este acceptabil pentru dumneavoastră să încălcați o normă legală/It is acceptable to break a legal rule if”.

### 2.2. Data Analysis

To carry out data processing in our study, we utilized IBM SPSS Statistics version 29.0.0.0, a powerful and widely used statistical software package. To assess the distribution of the data, we employed the Kolmogorov–Smirnov Test, a statistical test that determines whether or not a dataset is drawn from a normally distributed population. Additionally, we used the Cronbach’s alpha coefficient, a widely used measure of internal reliability, to evaluate the consistency of our measurement scale.

In order to ensure consistency with the original study, we employed a specific scoring methodology for our Likert scale. In this process, we assigned the lowest score of 1 to responses indicating “strongly agree”, while the highest score of 7 was assigned to responses indicating “strongly disagree”. By utilizing this approach, we ensured that our data analysis was consistent with the methodology of the original study.

## 3. Results

A total of 69 respondents completed the survey. The physicians who participated in our study worked in their profession for an average of 10.49 ± 8.27 years. Their mean age was 38.53 ± 8.28 years old. Table 2 provides a detailed summary of the baseline characteristics of the participating physicians, including specialty, work sector, geographic area, gender, and location.

The mean score obtained by physicians was 5.50 ± 1.15 out of a total score of 7. However, this score was found to be consistent across physicians of different genders (*p* = 0.119), ages (*p* = 0.395), and years of experience (*p* = 0.635). Furthermore, the scores’ distribution was similar across different work sector categories and specialties.

The physicians specializing in surgical specialties achieved a score of 5.82 ± 0.81, which was found to be significantly higher than the score obtained by physicians specializing in medical specialties, whose score was 5.50 ± 1.19. Out of all the items evaluated, Items 3, 5, and 1 received the highest scores. On the other hand, Items 10, 6, and 8 received the lowest scores (Table 3).

The internal consistency of the questionnaire was high, scoring a Cronbach’s alpha of 0.925. A Kolmogorov–Smirnov test determined that the data fit a normal distribution (*p* = 0.195). All inter-item correlations were scored positive, ranging from 0.127 to 0.809 (Table 4). The Kaiser–Meyer–Olkin Measure of Sampling Adequacy value of 0.881 and Bartlett’s test of sphericity with a *p*-value of < 0.0001 indicated adequate sampling.

## 4. Discussion

The present study validated the rule orientation scale for use in the Romanian language without any modifications to the original questionnaire. As a result, this scale can be employed not only to evaluate physicians’ attitudes toward breaking the law in certain situations but also by the general population and in various circumstances. The Romanian-translated scale has good internal consistency, and all inter-item correlations are good. The scores pertaining to the internal consistency of the questionnaire in our study were found to be comparable to those obtained in the original study [9].

The validated rule orientation scale in the Romanian language can be used to determine the conditions under which individuals find it acceptable to break the law. It provides a valuable tool for understanding the attitudes of the Romanian population toward ethical and legal dilemmas. By validating the rule orientation scale in the Romanian language, this study contributes significantly to the field of ethics research. It offers a reliable and valid method for assessing attitudes toward rule breaking, which can be used to better understand the ethical decision-making process in various contexts. Additionally, the scale’s high internal consistency suggests that it is a robust instrument that can be used confidently in future studies on this topic.

According to our study’s findings, Romanian physicians demonstrate high adherence to the law and only consider a limited number of circumstances where it may be acceptable to breach legal regulations. This is particularly noteworthy given previous studies indicating that less rule-oriented individuals are more likely to engage in misconduct [11,12,13]. People who are more likely to follow the law are likewise more likely to comply with it either because they have a moral drive toward law compliance or because they generally consider unacceptable law breaches [14].

Our study’s results indicate that gender, age, and years of experience were not significantly associated with physicians’ attitudes toward the acceptability of breaking the law. This is in contrast to the original study’s findings, which suggested that older adults tend to be more rule-oriented than their younger counterparts [9]. However, other studies have suggested that individuals may become more inclined toward engaging in illegal behavior as they age [11]. Various factors, including differences in study design, sample size, and population characteristics, may contribute to these inconsistencies in the literature. In addition, cultural, social, and economic aspects can influence individuals’ attitudes toward rule compliance and ethical-based decisions. Other factors, such as education, personal view of the legal system, and personal characteristics, can affect the will to obey the law [15].

Furthermore, our research also discovered that some physicians think it is acceptable to break the law under certain circumstances. Physicians received the lowest ratings in two of the twelve scenarios that could justify breaking the law. For instance, they consider that violating the law could be acceptable if they are unaware of its provisions or they could not comply with the law because they were unable to. The results suggest that physicians view legal infringements as acceptable if they experience difficulties in following the law, such as the inability to comply or a lack of awareness about it.

According to prior research conducted in the same area, physicians demonstrated a deficient level of legal understanding regarding informed consent and confidentiality. This can explain various legal breaches in the current hospital settings, and the lack of knowledge and the acceptability of legal breaches, in this case, can explain the low level of legal compliance with Romanian laws [7].

The study revealed the significance of the physicians’ professional and social surroundings in shaping their decision-making processes. When questioned about the impact of their colleagues’ and friends’ illicit conduct, the physicians exhibited remarkably elevated scores, indicating their reluctance to acknowledge the potential influence of such behaviors. Physicians were extremely hesitant to disregard the law due to the possible professional and social environmental impacts, particularly when their colleagues or acquaintances viewed such actions as permissible or were already violating the law. This may indicate a low level of peer influence within the hospital context. However, since the present study did not include residents, future research must investigate the effect of the work environment on resident physicians’ attitudes.

The present study is a pilot study with several limitations stemming from a relatively small sample size, a short survey period, and a restricted geographic exposure area.

Further research is necessary, utilizing additional measurement instruments, to examine the relationship between physicians’ rule orientation and their adherence to legal regulations. Such studies could provide additional insights into the factors contributing to low legal compliance levels among Romanian physicians, particularly concerning patients’ rights.

## 5. Conclusions

It is worth noting that while our study focused on physicians’ attitudes toward breaking the law, the validated rule orientation scale can also be used to explore the general population’s views on this issue. By employing this scale in future research, it may be possible to gain a better understanding of the factors that influence individuals’ ethical decision-making processes and to develop strategies to promote rule compliance across various sectors.

## Figures and Tables

**Table 1:** Rule Orientation—Romanian validated version of the scale.

Items	Answers *						
1. Dacă norma este în mod clar împotriva principiilor dvs. morale/The legal rule is clearly against your moral principles	1	2	3	4	5	6	7
2. Dacă această normă impune cerințe nerezonabile pentru dvs./This legal rule makes unreasonable demands of you	1	2	3	4	5	6	7
3. Dacă respectarea acestei norme este foarte costisitoare pentru dvs./Obeying this legal rule is very expensive for you	1	2	3	4	5	6	7
4. Dacă încălcarea ei este în general trecută cu vederea./This legal rule is not enforced	1	2	3	4	5	6	7
5. Dacă majoritatea colegilor și/sau prietenilor dvs. încalcă această normă./Most of your direct colleagues and/or friends also break this legal rule	1	2	3	4	5	6	7
6. Dacă sunteți, într-un fel sau altul, in imposibilitatea de a face ceea ce va cere aceasta normă./You are in one way or another unable to do what this legal rule asks	1	2	3	4	5	6	7
7. Dacă majoritatea colegilor și/sau prietenilor dvs. cred că încălcarea acestei norme este justificată./Most of your direct colleagues and/or friends think breaking the legal rule is justified	1	2	3	4	5	6	7
8. Dacă nu cunoașteți această normă./You do not know this legal rule.	1	2	3	4	5	6	7
9. Dacă nu înțelegeți această normă./You do not understand this legal rule.	1	2	3	4	5	6	7
10. Dacă această normă nu a fost încă publicată./This legal rule has not been published.	1	2	3	4	5	6	7
11. Dacă apreciați că această normă a fost elaborată fără a vă reprezenta interesele./You feel that this legal rule was made without representing your interests.	1	2	3	4	5	6	7
12. Dacă dvs. credeți că această normă juridică este pusă în aplicare în mod discreționar./You think this legal rule is enforced unfairly.	1	2	3	4	5	6	7

* 1 = acord total, 2 = ușor acord, 3 = acord, 4 = neutru, 5 = ușor dezacord, 6 = dezacord, 7 = dezacord total (eng.: 1 = fully agree, 2 = slightly agree, 3 = agree, 4 = neither agree nor disagree, 6 = disagree, 7 = fully disagree).

**Table 2:** Baseline characteristics of the physicians.

Variables		No	%
Specialty	Emergency	1	1.4
	Anesth. Intensive Care	1	1.4
	Non-surgical	33	47.8
	Surgical	20	29.0
	Dentists	5	7.2
	General Practitioners	1	1.4
	OBGYN	6	8.7
	Psychiatrists	2	2.9
Work sector	Both	24	34.8
	Private	22	31.9
	Public	23	33.3
Geographic area	Urban	69	100
	Rural	0	0.0
Gender	Female	40	58.0
	Male	26	37.7
	Dolj County	52	75.4
Location	Olt County	4	5.8
	Other	13	18.8

**Table 3:** Measurements from 12-Item Scale.

Item	Mean	SD
1. The legal rule is clearly against your moral principles	5.86	1.63
2. This legal rule makes unreasonable demands of you	5.74	1.67
3. Obeying this legal rule is very expensive for you	6.20	1.20
4. This legal rule is not enforced	5.84	1.36
5. Most of your direct colleagues and/or friends also break this legal rule	6.00	1.34
6. You are in one way or another unable to do what this legal rule asks of you	5.07	1.74
7. Most of your direct colleagues and/or friends think breaking the legal rule is justified	5.49	1.50
8. You do not know this legal rule	5.07	1.60
9. You do not understand this legal rule	5.70	1.32
10. This legal rule has not been published	4.01	2.02
11. You feel that this legal rule was made without representing your interests	5.67	1.44
12. You think this legal rule is enforced unfairly	5.39	1.58

**Table 4:** Measurements from 12-Item Scale.

Item	Corrected Item Total Correlation	Cronbach’s If Item Deleted
1. The legal rule is clearly against your moral principles	0.571	0.923
2. This legal rule makes unreasonable demands of you	0.708	0.917
3. Obeying this legal rule is very expensive for you	0.581	0.922
4. This legal rule is not enforced	0.846	0.913
5. Most of your direct colleagues and/or friends also break this legal rule	0.678	0.919
6. You are in one way or another unable to do what this legal rule asks of you	0.841	0.911
7. Most of your direct colleagues and/or friends think breaking the legal rule is justified	0.685	0.918
8. You do not know this legal rule	0.639	0.920
9. You do not understand this legal rule	0.606	0.921
10. This legal rule has not been published	0.555	0.927
11. You feel that this legal rule was made without representing your interests	0.749	0.916
12. You think this legal rule is enforced unfairly	0.835	0.912

## Data Availability

Data are available, on request, from the corresponding author.
